# Phosphorylated *Radix Cyathulae officinalis* Polysaccharides Act as Adjuvant via Promoting Dendritic Cell Maturation

**DOI:** 10.3390/molecules22010106

**Published:** 2017-01-10

**Authors:** Haibo Feng, Sean P. McDonough, Jing Fan, Shiping Yang, Xuelian Zhao, Yong Lu, Yun Gan, Xiao Yi, Yung-Fu Chang

**Affiliations:** 1 Department of Veterinary Medicine, Southwest University, Rongchang, Chongqing 402460, China; spyswu@sina.com (S.Y.); fenghb1981@126.com (X.Z.); luyongswu@163.com (Y.L.); umbrella_fj@163.com (Y.G.); yixiaoswu@163.com (X.Y.); 2 College of Veterinary Medicine, Cornell University, Ithaca, NY 14850, USA; spm13@cornell.edu; 3 Sichuan Industrial Institute of Antibiotics, Chengdu University, Chengdu 610051, China; fanjingcdu@sina.com

**Keywords:** *Radix Cyathulae* polysaccharide, phosphorylation modification, adjuvant, dendritic cell

## Abstract

The aim of this study was to investigate whether phosphorylated *Radix Cyathulae officinalis Kuan* polysaccharides (pRCPS) used as adjuvant with foot-and-mouth disease vaccine (FMDV) can stimulate specific humoral and cellular immune responses in ICR mice. The results demonstrated that pRCPS significantly up-regulated FMDV-specific IgG, IgG1, IgG2b and IgG2a antibody levels and splenocyte proliferation. pRCPS also promoted the killing activities of cytotoxic T lymphocytes (CTL) and natural killer cells (NK). In addition, pRCPS enhanced the expression levels of IL-2, IL-4, and IFN-γ in CD4^+^ T cells and the level of IFN-γ in CD8^+^ T cells. Importantly, pRCPS enhanced the expression of MHCII, CD40^+^, CD86^+^, and CD80^+^ in dendritic cells (DCs). This study indicated that phosphorylation modification could increase immune-enhancing activities of RCPS, and pRCPS could promote humoral and cellular immune responses through facilitating DC maturation.

## 1. Introduction

Polysaccharides are polymeric carbohydrate molecules formed by many monosaccharide units linked by glycosidic bonds. They are widely distributed in the cell membranes of higher plants, algae, bacteria fungi, and animals. Polysaccharides extracted from natural plants have been used as novel adjuvant with low toxicity, low side effects, and stimulatory activities [1,2,3,4]. These natural polysaccharides used as adjuvants can effectively activate cellular and humoral immunity in the host [5,6,7,8].

In recent years, studies have shown that polysaccharides display excellent immune-enhancing activity. It is well known that biological activities of polysaccharides depend on their structural characteristics, namely the glycosidic bond of the main chain sugar subunits [9,10]. Molecular modification of natural polysaccharides can significantly promote their immune-enhancing activity [10,11,12]. Presently, phosphorylation modification of polysaccharides is a commonly used technique to modify the sugar. Many researchers reported that phosphorylation modification of polysaccharides can modulate the immune-enhancing activity [13,14,15,16,17]. For example, Phosphorylation of dextran (P-Dex) with a pathogen-associated molecular pattern (PAMP) can trigger B cell proliferation, increase cytokine production, promote antitumor activity and induce dendritic cell (DC) maturation in splenocytes [13,14,15,16]. Oral administration P-Dex can significantly enhance the specificity of immunological responses in ovalbumin-immunized mice [16]. Furthermore, Nagasawa et al. (2010) have demonstrated that phosphorylated dextran (P-Dex) can improve the immunological response to specific antigens [18].

*Radix Cyathulae officinalis Kuan* is a perennial herbaceous plant widely distributed in tropical areas of Asia and Africa. Their roots are a commonly-used Chinese traditional herbal medicine to enhance immune functions in humans and animals [19,20,21,22,23]. In our previous studies, *Radix Cyathulae officinalis Kuan* polysaccharide (RCPS), which was isolated by water decoction and ethanol precipitation, dramatically increased both specific and non-specific immune responses [19,20,21].

In the present study, the RCPS were extracted and purified using water decoction and ethanol precipitation methods as previously described [19]. Subsequently, we adapted a previously reported method for the chemical phosphorylation of RCPS to pRCPS [24], and the preliminary structural characterization of the pRCPS was then determined by physicochemical properties, scanning electron microscopy (SEM) analysis, and infrared (IR) spectroscopy. In addition, ICR mice vaccinated with FMDV with pRCPS as an adjuvant were evaluated for antigen-specific antibody titer, splenocyte proliferation, T helper (Th) cytokine expression, NK cells, CTL activity, and DC maturation. The purpose of this research was to evaluate the use of phosphorylation modification of RCPS to improve the immune-enhancing activity in mice.

## 2. Results and Discussions

### 2.1 Results

#### 2.1.1. Chemical Properties of pRCPS

The physicochemical properties of pRCPS were determined. The color of pRCPS was light brown. The solubility test suggested that the pRCPS was water soluble. The results of the phenol–sulfuric acid tests (+) suggested that the pRCPS was a kind of sugar. α-naphthol tests (+) revealed that the pRCPS is carbohydrates. Iodination tests (−) revealed that pRCPS did not contain starch. The Fehling’s tests (−) suggested that the pRCPS did not contain reducing sugar. The carbazole tests (+) revealed that pRCPS contained some uronic acid. FeCl_3_ tests (−) suggested that pRCPS did not contain phenol. The full wavelength scanning (−) analysis and Coomassie brilliant blue tests (−) revealed that pRCPS did not contain proteins. Taken together, the extractions were polysaccharides and contained some uronic acid, but did not contain starch, proteins, reducing sugar, or polyphenol.

The molecular weight (MW) of RCPS and pRCPS was determined to be 181.8 kDa and 212.9 kDa, respectively. Using the molybdenum blue spectrophotometry method, and with potassium dihydrogen phosphate as a standard, the phosphate graft quantity of the pRCPS were measured to be 9.52%. Using the anthrone-sulfuric acid method, the polysaccharide content (*w*/*w*) of the pRCPS was determined to be 97.3%. Using the carbazole-sulfuric acid method, the uronic acid content of pRCPS was determined to be 6.42%. Using the phenol–sulfuric acid method, the polysaccharide samples were found to not be contaminated with proteins. The composition of monosaccharide pRCPS was determined by GC-MS; pRCPS mainly consisted of glucose (49.1%), galactose (16.1%), rhamnose (14.4%), mannose (9.8%), arabinose (6.4%), and fructose (4.2%), respectively.

#### 2.1.2. FR-IR Spectroscopy Analysis

**Figure 1:**
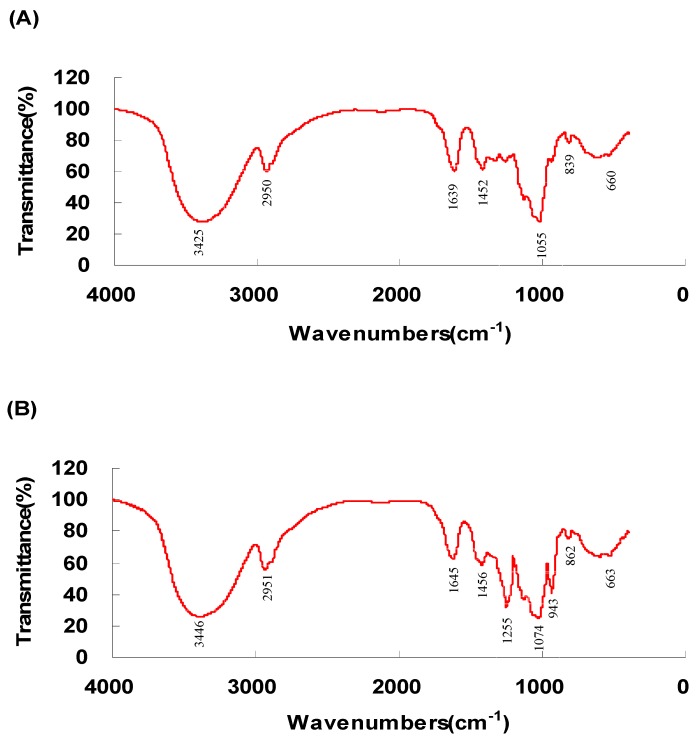
FT-IR spectra of pRCPS. The IR spectrum of the pRCPS powder was analyzed using the potassium bromide pellet method in an IR spectrophotometer. The data were recorded between 4000 and 400 cm^−1^. The IR characteristics of: RCPS (**A**); and pRCPS (**B**) are illustrated.

The FT-IR spectra of the RCPS and the pRCPS determined between the 400 to 4000 cm^−1^ range are illustrated in Figure 1A,B. In the 1000 to 1200 cm^−1^ region, RCPS had a specific absorption band, which was dominated by (C-OH) stretching vibrations of side groups and ring vibrations overlapped with the (C-O-C) glycosidic band vibrations. The absorption peak between 1500 and 1700 cm^−1^ corresponded to the C=O asymmetric stretching vibration, and the characteristic absorption of weak C-H stretching vibrations band was observed in the 2800 to 3000 cm^−1^ region. The broadly intense stretched peak in the 3200 to 3600 cm^−1^ region was attributed to the O-H stretching vibrations, In addition, obvious characteristic absorption peaks of polysaccharides can be observed in the spectra of RCPS and pRCPS. The FRIR spectroscopy result indicated that the RCPS and the pRCPS were polysaccharides. Compared with the spectrogram of RCPS, the FT-IR spectroscopy of pRCPS showed two obviously absorption peaks, one peak around at the 1000 to 800 cm^−1^ region, representing a symmetrical C-O-P vibration described a C-O-PO_3_ group, and another at the 1300 to 1200 cm^−1^ region that was attributed to an asymmetrical P=O stretching vibration, which reveals that the pRCPS was successfully synthesized [25].

#### 2.1.3. Effects of pRCPS on FMDV-Specific IgG and the IgG Subclasses

**Figure 2:**
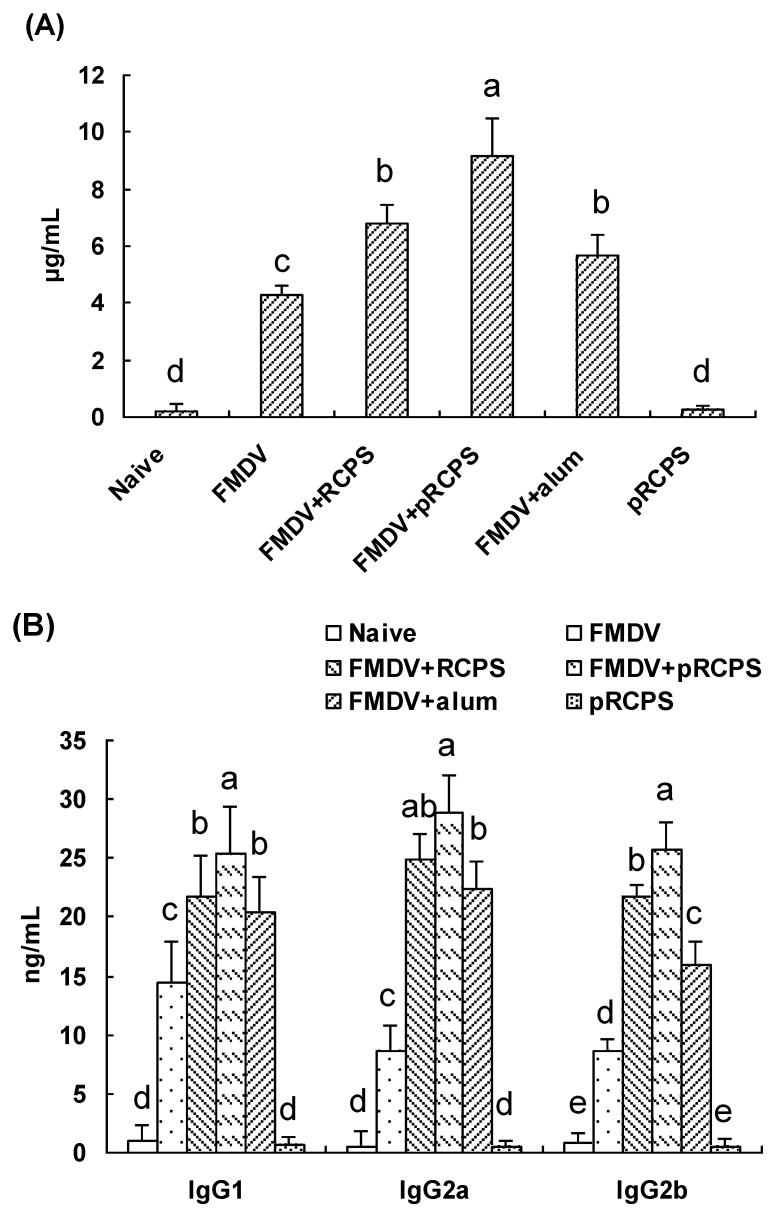
Effect of the pRCPS on antibody level. Blood samples were collected and serum was prepared from immunized mice in all groups on Day 14 after the second immunization for ELISA. The FMDV-specific IgG (**A**) and IgG2a, IgG1, and IgG2b (**B**) were determined by ELISA as described in Experimental Section. The concentration of IgG and IgG isotopes are presented as mean ± standard deviation. The different letters on a column differ significantly (*n* = 10, *p* < 0.05).

Serum samples of mice were collected 14 days after second boost, and allowed to clot for 2 h at 37 °C. The FMDV-specific IgG and the IgG isotypes were determined using indirect ELISA. The result shows the antigen-specific IgG antibody titer in pRCPS groups were dramatically higher than in RCPS group, alum group, FMDV group, and naive group (*p* < 0.05) (Figure 2A). The antibody level in FMDV + RCPS group and alum group were significantly higher than in the FMDV group and in naive group (*p* < 0.05). In addition, all IgG subclass antibody levels in pRCPS groups were significantly higher than in the other group (*p* < 0.05) (Figure 2B). Our experiment revealed that the pRCPS dramatically promoted antigen-specific antibody levels in the FMDV-immunized ICR mice.

#### 2.1.4. Effects of pRCPS on Splenocyte Proliferation

**Figure 3:**
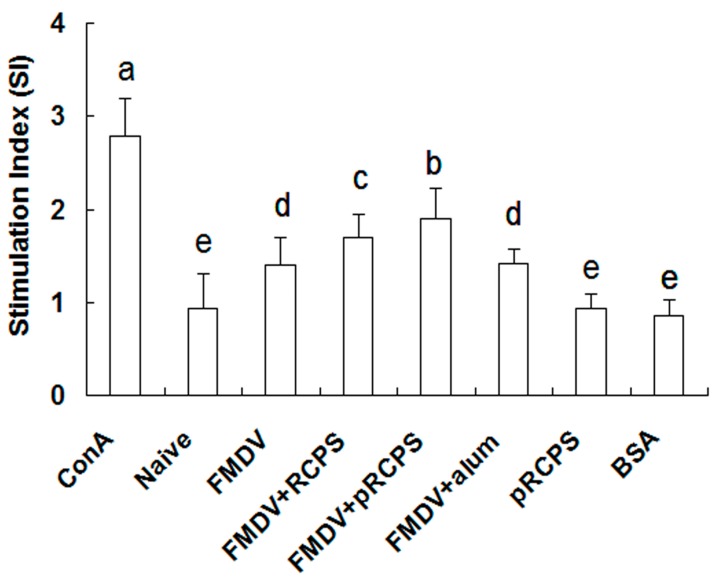
Effect of pRCPS on splenocytes proliferation in vitro. To investigate the effect of pRCPS on splenocyte proliferation in vitro, splenocyte were isolated from ICR mice in all groups on Day 14 after the second booster. splenocyte proliferation assay was performed by the CCK-8 method as described in Experimental Section. BSA as negative control, and ConA as positive control. The proliferation was expressed as stimulated index (SI). The different letters on a column differ significantly (*n* = 10, *p* < 0.05).

Splenocyte proliferation results show the splenocyte proliferation levels in the pRCPS groups were remarkably higher than the RCPS group, alum group, FMDV group, naive groups, and BSA groups (*p* < 0.05) (Figure 3). In this experiment, pRCPS employed as an adjuvant dramatically enhanced splenocyte proliferation levels compared to FMDV group.

#### 2.1.5. Effects of pRCPS on Helper T Cells

T helper (Th) cytokine expression is a key indicator that reflects the immune response levels in an animal. Single splenocyte suspensions prepared on Day 14 after the second boost were cultured with the FMDV antigen. In order to detect the effects of pRCPS on antigen-specific Th cell response, cytokine expression level in CD4^+^ T cells were detected through intracellular staining. As shown in Table 1, IL-2, IL-4, and IFN-γ expression level of CD4^+^ T cells in the pRCPS group were numerically higher than in the RCPS group, FMDV group, alum group, and naive group (*p* < 0.05). As expected, alum also enhanced IL-4 expression, but this enhancement was less than the pRCPS group and RCPS group. In addition, IFN-γ expression in CD8^+^ T cells from mice immunized with FMDV plus pRCPS was dramatically higher than in the other groups (*p* < 0.05). These results demonstrated that pRCPS significantly stimulate Th cells to secrete Th1- and Th2-type cytokines, revealing that pRCPS can promote both Th1 and Th2 responses simultaneously.

#### 2.1.6. Effects of pRCPS on Cytotoxic T Lymphocyte (CTL)

**Figure 4:**
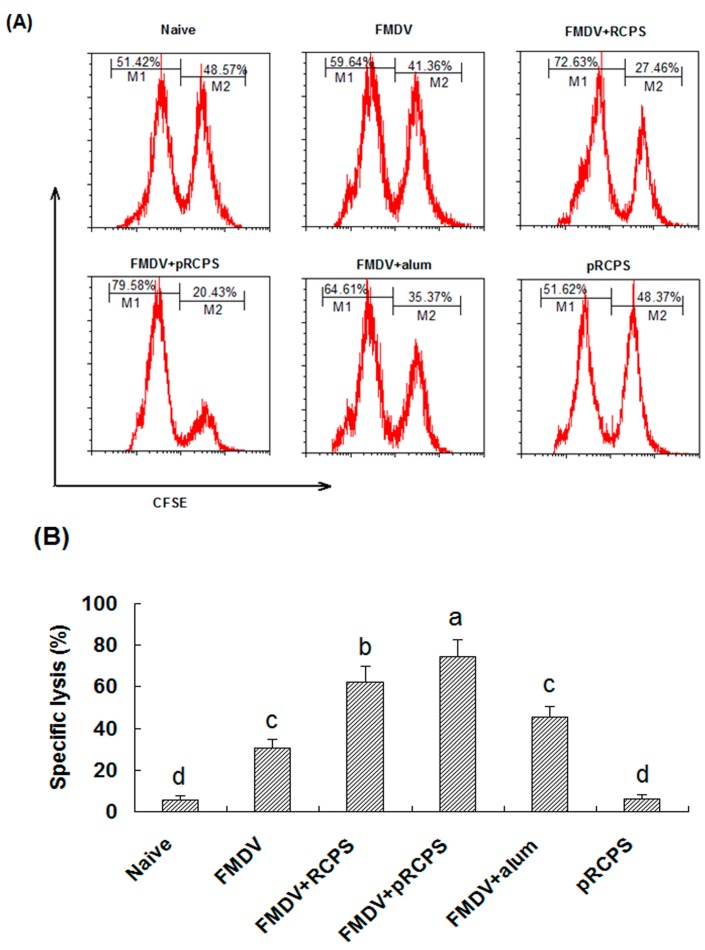
Effects of pRCPS on the FMDV-specific cytotoxicity in vivo. The effect of pRCPS on FMDV-specific cytotoxicity were evaluated in vivo, CFSE labeled target cells (CFSE^high^ and CFSE^low^ at 1:1 ratio) were prepared and injected intravenously into FMDV-immunized mice on Day 14 after the second booster. After four hours, the mice were sacrificed, spleens were removed and single cell suspensions of splenocytes were prepared, and ratio changes between the CFSE^low^ (M1) and CFSE^high^ (M2) target cell populations were determined. The percentage of antigen-specific lysis were showed in Figure 4A,B. The lysis percentage was calculated for each group as described in experimental section. The different letters on a column differ significantly (*n* = 10, *p* < 0.05).

It is well known that CTL responses play an important part in the protection against viruses and other intracellular pathogens. The effects of pRCPS on CTL responses show the percentage of FMDV-specific lysis of the target cells in mice in the FMDV + pRCPS groups was 74.33% (Figure 4A,B). The percentage of lysis in the RCPS, alum, FMDV and naive groups was 62.20%, 45.26%, 30.66%, and 5.55%, respectively. Our results showed that pRCPS significantly promoted FMDV-specific cytotoxic responses compared to RCPS group, alum group, FMDV group, and naive group (*p* < 0.05), suggesting pRCPS can significantly increase CTL activity.

#### 2.1.7. Effects of pRCPS on Killing Activity of NK Cell

**Figure 5:**
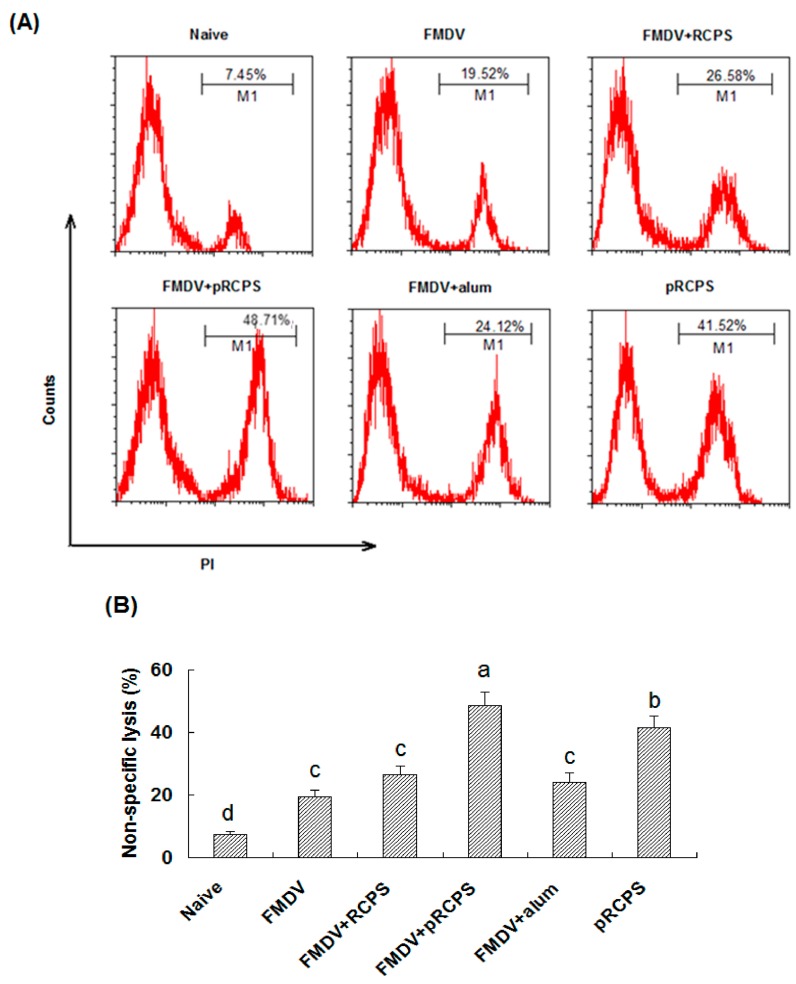
Effects of pRCPS as an adjuvant on the activity of NK cells. In order to evaluate the effect of pRCPS on the killing activity of NK cells in vitro, K562 cells labeled with CFSE served as target cells while splenocytes prepared from FMDV-immunized ICR mice in all groups were used as effector cells. After killing 4 h, the PI was added to the reaction mixture. Then, the killing activity of NK cells was analyzed by FACS. The CFSE and PI double positive cells were selected and recorded, and the percentage of CFSE and PI double stained cells in total CFSE positive cells represent the non-specific lysis percentage. The percentage of non-specific lysis were showed in Figure 4A,B. The percentage lysis was determined for each group as described in Experimental Section. The different letters on a column differ significantly (*n* = 10, *p* < 0.05).

The killing effects of splenocytes against K562 cells was measured by FACS. As shown in Figure 5A,B, the percentage of non-specific lysis of the target cells in mice in the FMDV + pRCPS groups and pRCPS groups was 48.71% and 41.52%. The percentage of lysis in the RCPS, alum, FMDV and naive groups was 26.58%, 24.12%, 19.52%, and 7.45%, respectively. Our result showed that pRCPS significantly enhanced the killing activity of NK cells in FMDV vaccinated mice (*p* < 0.05), and reveal that pRCPS could enhance the killing activity of NK cells.

#### 2.1.8. Effects of pRCPS on DCs

**Figure 6:**
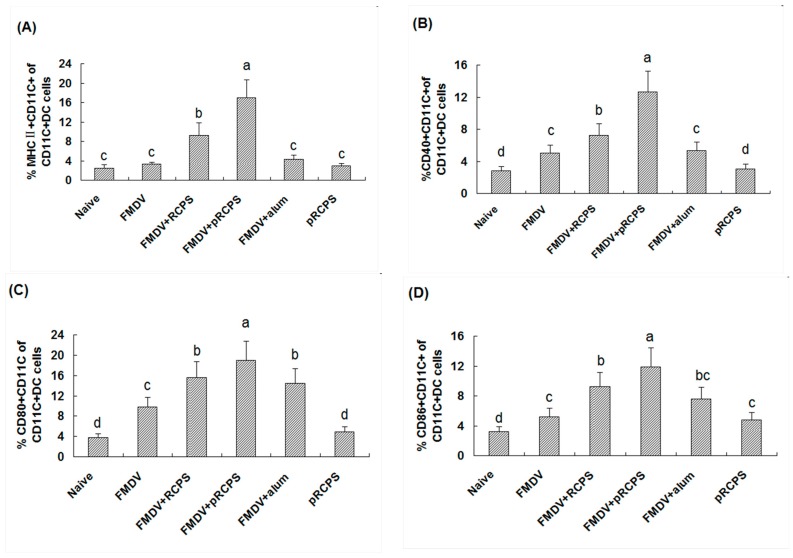
Effect of pRCPS as an adjuvant on the maturation of DCs. The spleens were removed from immunized mice on Day 3 after the first immunization, and the splenocytes suspensions were prepared. The cells were double stained with anti-CD11c-FITC and anti-MHC II-PE, anti-CD11c-FITC and anti-CD80-PE, anti-CD11c-FITC and anti-CD86-PE, or anti-CD11c-FITC and anti-CD40-PE respectively. Then, the expression levels of surface molecular (MHC II, CD40^+^, CD80^+^, and CD86^+^) were analyzed using FACS. FITC stained positive cells were represent DC cells, The PE stained positive cells are represent the cells which expressed surface molecular. The PE and FITC double positive cells identified as those DCs which expressed surface molecular. The percentage of PE and FITC double positive cells in total cells were selected and recorded in each sample. The percentage of MHC II, CD40^+^, CD80^+^, and CD86^+^ in total DCs are shown in (**A**–**D**). The values are presented as mean ± standard deviation. The different letters on a column differ significantly (*n* = 10, *p* < 0.05).

The expression frequency of MHC II, CD40^+^, CD86^+^, CD80^+^ in DCs cells are important indicators can reflect the mature levels of DCs. Thus, the effects of pRCPS on the maturity of DCs in mice were investigated on Day 3 after the first immunization. The frequency of MHC II, CD80^+^, CD40^+^, CD86^+^ expression levels on DCs cells in the pRCPS group were remarkably higher than in all other groups (Figure 6). The experiment results revealed that pRCPS activates DCs and induces DC maturation, and the activity of pRCPS activated the DCs is stronger than the RCPS.

### 2.2. Discussion

Humoral immune response mediated by B lymphocytes is one of the major indicators of resistance of some infectious diseases, and the antibody level of the blood is a key biomarker reflect the level of humoral immune response. In general, if the antibody titer is higher, the infection will be less serious [26,27,28]. Molecular modifications of polysaccharide have been shown to induce a higher humoral immune response. Qiu et al. (2014) reported that selenylation modified garlic polysaccharide (sGPS) could greatly increase serum antibody titers in chickens immunized with Newcastle disease (ND) vaccine, and antibody levels in the sGPS group were significantly higher than the GPS group [29]. Zhang et al. (2013) also confirmed that the sulfated ophiopogonpolysaccharide (OPS) and jujube polysaccharide (JPS) significantly enhanced the serum antibody level response to ND vaccine [24]. Our experiment result revealed that antigen-specific IgG, IgG2a, IgG1, and IgG2b titers in pRCPS + FMDV group were significantly higher than in the RCPS, alum, FMDV, and naive groups. Comparisons between the phosphorylating and non-phosphorylating groups demonstrate that the humoral immune-enhancing efficacy of these pRCPS were significantly stronger than that of non-phosphorylating RCPS.

Cellular immune function can regulate immune responses by accelerating the clearance of intracellular pathogens, and by secreting cytokines to against infection [30]. Lymphocyte proliferation is a key factor that can reflect the level of cellular immunity. The present result suggested that the proliferation level in the pRCPS groups was significantly higher than in the RCPS, alum and FMDV groups. This finding revealed that phosphorylated modification could improve the immune-enhancing activity of RCPS. This is consistent to other studies that show phosphorylated polysaccharides could enhance lymphocyte proliferation [15,17].

It is well known that Th1 or Th2 responses can eliminating the virus and preventing its entry into the host, and Th responses which can be generated by antigenic stimulation and modulated by T-cell cytokines [31,32]. Type 1 helper T (Th1) cells can induce cell-mediated immunity by secreting Th1 cytokines, such as IFN-γ, IL-2, and tumor necrosis factor TNF-α which stimulates the maturation of DC and regulates the proliferation and division of lymphocytes. The type 2 helper T (Th2) cells can induce humoral immune responses by secreting Th2 cytokines, such as IL-13 IL-10, IL-4, induce a strong antibody response and perpetuates the Th2-biased immune response [33,34]. An ideal adjuvant can stimulate both Th1 and Th2 responses to induce protective immunity against certain infectious diseases is desirable. Nagasawa et al. (2010) have reported that phosphorylated dextran (P-Dex) functions as an adjuvant that can modulate immune responses by maintaining a balance between Th1 and Th2 cells in vitro [18]. Kawashima et al. (2012) have also demonstrated that fucoidan as a sulfated polysaccharide can enhance an immunobalanced Th1/Th2 immune response in ovalbumin-immunized mice, as indicated by the significant production of both Th1/Th2 cytokines [35]. In this study, we also determined the expression of Th1/Th2 cytokines by CD4^+^ T cells and CD8^+^ T in FMDV-immunized mice. The results revealed that pRCPS can act as an adjuvant that significantly increases the expression levels of IFN-γ, IL-2, and IL-4 in CD4^+^ T cells and IFN-γ in CD8^+^ T cells (Table 1). In addition, pRCPS can significantly increase the IgG1, IgG2b, and Ig2a levels in FMDV-immunized mice. The present result revealed that pRCPS significantly promoted both Th1 and Th2 immune responses.

Cytotoxic lymphocytes (CTLs and NK cells) are two major populations of lymphcoytes [36,37,38,39,40] CTLs belong to the CD8^+^ subset of T cells, which express T-cell receptors (TCRs) that can recognize, attack and lyse a cells infected with specific pathogens. NK cells are part of the innate immune system, unlike CTL, which are mediators of adaptive immunity, and the elimination of pathogens by NK cells is non-specific [41]. CTL and NK cell activity assays are commonly used to evaluate cellular immune response levels. In our study, pRCPS as an adjuvant could enhance the lysis activities of NK cells and CTL from splenocytes in FMDV-immunized ICR mice (Figure 4 and Figure 5), suggesting that phosphorylation modification of RCPS can enhance antigen-specific and non-specific killing activities against pathogens.

DCs as a kind of professional antigen presentation cells (APC) that can capture and process antigens, and proteases to initiate an immune response [42,43]. The effect of DCs to modulate immunity is dependent on DC maturation. Only mature DCs can activate the adaptive immune cells during the invasion of an exogenous pathogen [44]. Numerous factors can facilitate maturation following antigen uptake and processing within DCs. In general, the process of DC maturation involves a redistribution of MHC I/II from intracellular endocytic compartments to the DC surface, up-regulation of co-stimulatory molecules on the surface, down-regulation of antigen internalization, cytoskeleton re-organization, production of cytokines, chemokines and proteases, and surface expression of adhesion molecules and chemokine receptors. The expression of MHC molecules and co-stimulatory molecules (CD80^+^, CD40^+^, and CD86^+^) on DCs can indicate DCs mature level, and correlate with T lymphocyte differentiation [45]. Recently, many studies have demonstrated that natural polysaccharides can facilitate DCs maturation. Shin et al., 2013 reported that a soluble polysaccharide was extracted from *Morifructus* (MFP), and the effects of MFP on DCs included significant up-regulation of the expression of MHC I/II, CD40^+^, CD80^+^, CD86^+^ on DCs, and promoted the production of IL-12, TNF-α, IL-1b, and IFN-β. These results suggested that MFP induces murine DCs and leads to maturation [46]. Other studies have also demonstrated that *Astragalus* polysaccharide used as an adjuvant significantly enhanced immune responses in mice by activation of DCs and lead to their maturation [47,48]. In our study, pRCPS could enhance the expression level of MHC II, CD40^+^, CD86^+^, CD80^+^, indicating that pRCPS can act as an adjuvant leading to the maturation of DCs and enhanced antigen uptake activity of DCs in FMDV-immunized ICR mice.

Many other researchers investigated the mechanism of polysaccharide induce the DCs maturation [46]. Shin et al. reported that MFP promoted phosphorylation of mitogen-activated protein kinase (MAPKs), and nuclear translocation of (NF-κB p65) subunit, suggesting that MFP could induce DC maturation via activated important TLR-4 downstream signal molecules. In our previous experiment, RCPS enhanced the immune response through TLR-2 and TLR-4 signal pathways [46]. In the present study, pRCPS increased the cellular and humoral immune responses and induced DCs maturation in FMDV immunized mice. The possible mechanism of pRCPS facilitating DCs may through TLR-2/4 signal pathway. We will investigate the precise mechanism in further study.

## 3. Experimental Section

### 3.1. Reagents and Cell Line

Acetic acid and NaBH_4_ was obtained from Sinopharm Group Co., Ltd. (Shanghai, China). A carboxyfluorescein succinimidyl ester (CFSE) kit was obtained from Fanbo Biochemicals (Beijing, China). Anti-mouse fluorescent-labeled monoclonal antibodies (anti-CD8-PE, anti-CD4-PE, anti-IL-4-PE, anti-CD4-FITC, anti-IFN-γ, IL-2-FITC, anti-MHCII-PE, anti-CD40-PE, anti-CD80-PE, anti-CD11c-FITC and anti-CD86-PE) were purchased from eBiosciences (San Diego, CA, USA). Goat anti-mouse IgG, IgG2b, IgG2a and IgG1 peroxidase conjugates were purchased from Santa Cruz Biotechnology Inc. (Santa Cruz, CA, USA). RPMI-1640 medium, Concanavalin A, and propidium iodide were purchased from Sigma Chemical Co. (Saint Louis, MO, USA). K562 cell lines were purchased from the Institute of Cell Biology, Chinese Academy Sciences (Shanghai, China). All other reagents were chemicals of analytical grade.

### 3.2. Extraction and Phosphorylated Modification of Polysaccharide

The extraction and purification of RCPS was performed as previously described [18]. Briefly, the dry powder of RC was extracted in water three times under reflux. The aqueous extract was filtered, centrifuged at 6000× *g* for 10 min and the supernatant was precipitated with 95% ethanol at 4 °C for 12 h. The precipitate was then dissolved in distilled water, protein removed by the Sevag method, dialyzed in a dialysis sack against distilled water for 48 h, and then precipitated with 95% ethanol for 12 h at 4 °C. The resulting precipitate was centrifuged at 6000× *g* for 10 min, washed three times with 85% ethanol, and the precipitate was dried and collected. Finally, the precipitate was permeated through a macroporous adsorption resin (ADS-7) to eliminate other pigments, followed by elution through a DEAE Sephadex™ A-25 column to separate it from the other carbohydrates. The collected eluates were concentrated and lyophilized to produce the total RCPS fraction. The carbohydrate content (*w*/*w*) of RCPS was 96.6%, as determined by the phenol-sulfuric acid method [11].

Purified RCPS was phosphorylated according the Zhang’s method [23], with a minor modification. Briefly, pRCPS was prepared using the following steps. Sodium tripolyphosphate and sodium trimetaphosphate complexes in 1:4 (*w*/*w*) ratios were prepared. Subsequently, RCPS was added to the complex at 0.01 g/mL, pH 9 with constant stirring and incubated at 85 °C for 7 h. The reaction solution was cooled to room temperature, precipitated by adding 1:4 (*v*/*v*) ratio 95% ethanol at 4 °C for 24 h and then lyophilized. Next, the compound was re-dissolved with water, and dialyzed in a dialysis sack with a 14,000 cut-off molecular weight against distilled water until conductivity decreased from 1 × 10^4^ μs/cm to 160 μs/cm. Each preparation was lyophilized again and called pRCPS [49].

### 3.3. Characterization of RCPS and pRCPS

#### 3.3.1. Physicochemical Property Analysis

We determined the physicochemical properties of RCPS and pRCPS using the following methods: color observation, iodination reaction, solubility test, phenol-sulfuric acid test, α-naphthol test, uronic acid carbazole reaction, Fehling’s test, full wavelength scanning, FeCl_3_ test, and Coomassie brilliant blue test as previously described [50].

#### 3.3.2. Analysis of Monosaccharide Composition, Uronic Acid, Content of Carbohydrate and Protein of pRCPS

The hydrolysis method was used to measure the monosaccharide composition of pRCPS by gas chromatography (GC) analysis as previously described [50]. Twenty milligrams pRCPS was hydrolyzed using 2 M trifluroacetic acid for 6 h at 100 °C to hydrolyze and release the component monosaccharides. The monosaccharide composition of RCPS was determined using GC-MS alditol acetates of standard monosaccharides (l-rhamnose, d-glucose, d-fructose, d-xylose, d-galactose, d-arabinose, and d-mannose) with inositol as the internal standard [51].

The molecular weight (MW) of RCPS and pRCPS was measured using gel permeation chromatography (GPC) on a column (60 cm × 1.6 cm) of Sephadex G-100. The column was eluted by ultrapure water at a flow rate of 0.6 mL/min. The average molecular weight was detected by Dextran standards (molecular weights: 11,600; 48,600; 80,900; 147,600; 273,000; 667,800; and 1,185,000).

The protein content was determined by the Bradford method [52] using bovine serum albumin as a standard. The uronic acid content of pRCPS was measured by using the carbazole-sulfuric acid method, with glucuronic acid as a standard [53]. The content (*w*/*w*) of polysaccharide was detected by the phenol-sulfuric acid method with glucose as a standard [54].

#### 3.3.3. Analysis the Phosphate Graft Quantity

The phosphate graft quantity of pRCPS was determined by the molybdenum blue spectrophotometry method [55]. Briefly, 0.5 mg/mL of the phosphate standard solution was prepared by mixing distilled water with 50 mg potassium dihydrogen phosphate and then adding 0.1 mL, 0.2 mL, 0.3 mL, 0.4 mL, 0.5 mL, 0.6 mL, 0.7 mL of the phosphate standard solution to different 50 mL volumetric flask. Next, to each flask was, added 2.0 mL 15% sulfuric acid solution, 2.0 mL 5% ammonium molybdate solution, 2.0 mL 0.5% hydroquinone solution and 2.0 mL 2% sodium sulfite solution. Thereafter, distilled water was added to 50 mL, the solution heated in a water bath at 30 °C for 20 min, cooled to room temperature, and the absorbance of phosphate standard solution was determined at 660 nm and a standard curve and equation was obtained.

1 mL of concentrated nitric acid and 1 mL concentrated sulfuric acid was added to a beaker, and then 0.5 g pRCPS was added and heated it until produce smoke. Next, 1 mL 30% hydrogen peroxide solution was added, the mixture cooled, and then reheated, repeating the previous steps until no smoke was produced in the beaker and the solution became yellow or colorless and transparent. After cooling, 1 mL 6 mol/L dilute hydrochloric acid was added, and heated the solution until no smoke was produced. Subsequently, the reaction solution was transferred to a 50 mL volumetric flask, and the absorbance of the reaction solution was measured according to the standard curve method, and the content of phosphate was calculated according the regression curve. The phosphate content of RCPS was calculated using the same method as the control group.

#### 3.3.4. Infrared Spectroscopy Analysis of pRCPS

In this study, the organic functional groups of pRCPS were detected using Infrared (IR) spectra (FTIR-8400S, Shimadzu Co., Kyoto, Japan), and the purified RCPS and pRCPS was dried and ground with KBr powder and pressed into pellets for FT-IR measurement. The IR characteristic of pRCPS between the range of 4000 cm^−1^ and 400 cm^−1^ were measured.

### 3.4. Endotoxin Detection

The pRCPS was diluted to 1 mg/mL with physiological saline, and sterilized by pasteurization. The endotoxin concentration was tested by pyrogen test; when the endotoxin amount reached the standard of the Chinese Veterinary Pharmacopoeia (<0.5 EU/mL) [56], the pRCPS solution was stored at 4 °C for the test.

### 3.5. Animal Vaccination

Institute of Cancer Research (ICR) female mice (weighing 18–22 g, 5 weeks old, Grade II) were obtained from Sichuan Laboratory Animal Center (SLAC) Co. Ltd. (Sichuan, China). In this study, the procedures related to animal care were performed in accordance with the internationally accepted principles as found in the Guidelines for Keeping Experimental Animals issued by the government of China and is approved by the IACUC of Southwest University. The pRCPS was injected once a day for three consecutive days before each immunization. The mice (10 per group) were vaccinated subcutaneously twice, at two-week intervals, with different vaccine formulations, the animals groupings and their vaccinations, including vehicle controls, are listed in Table 2.

### 3.6. FMDV-Specific Antibody ELISA

The serum was collected 14d after the booster vaccination, and the FMDV-specific IgG titer and IgG isotypes were measured using the indirect double-antibody sandwich enzyme-linked immunosorbent assay (ELISA) as described previously [57,58]. The optical density (OD) was measured by an ELISA reader (Model 680, Bio-Rad, Philadelphia, PA, USA) at 450 nm. The concentrations for total IgG, IgG isotypes were measured according to optical density values of the diluted samples on the standard curve, which was plotted by the matched mouse IgG protein with serial dilutions (in steps of two fold) from 20 to 0.01 ng/mL and multiplying by the dilution factor (100-fold).

### 3.7. Analysis Proliferation Activity of Splenocytes

The spleens were removed on Day 14 after the boost. Single cells suspensions were prepared according to previous report [18]. Splenocytes numbers were counted by Trypan blue dye exclusion method. Cell viability exceeded 95%. The splenocyte proliferation level was measured by Cell counting Kit-8 (Beyotime, Haimen, China) according to the manufacturer’s instructions [18,20]. The optical density (OD) was determined by a microplate reader (Model 680, Bio-Rad) at 450 nm. The stimulation index (SI) was calculated according to the following formula: SI = the OD value of the stimulation-cultures/the OD value of non-stimulated cultures.

### 3.8. Analysis Killing Activity of CTL Cell by Flow Cytometry (FCM)

The killing activity of CTL was performed as previously reported [59,60]. A single cell suspension of splenocytes prepared from spleen of naive ICR mice and was split equally into two portions. One portion was labeled with CFSE solution (0.25 μM) as the control, named CFSE^low^ cells. An equal fraction of splenocytes was incubated with FMDV antigen solution, and labeled using the CFSE solution (2.5 μM) as target cells, named CFSE^high^ cells. The target cells and control cells were mixed together in a ratio of 1:1. The mixture cells suspension was injected into immunized ICR mice on Day 14 after the second boost at 2 × 10^7^ cells each mice through the tail vein. Four hours after injection, the spleens of injected mice were collected, and splenocyte suspensions were prepared. Subsequently, cell suspensions were analyzed using a FACS Calibur analyzer (BD Biosciences, San Jose, CA, USA). Specific-lysis was calculated using the following equation: Killing ratio = percentage CFSE^low^ divided by the percentage of CFSE^high^; The specific-lysis percentage = (1 − (killing ratio unprimed/killing ratio primed) × 100).

### 3.9. Analysis Killing Activity of NK Cell by Flow Cytometry (FCM)

The spleens were removed from immunized ICR mice on Day 14 after second immunization, and the single cell suspensions were prepared and were used as effector cells. K562 cells stained with CFSE solution were used as target cells. The cytotoxic activity of NK cells from spleen was performed as described by previous reported [18,61]. The final cell suspension was then analyzed using FACS Calibur (BD Bioscience). The CFSE stained cells were represented as the total target cells; the PI and CFSE double stained cells were identified as dead cells. The percentage of double stained cells (CFSE and PI) in total CFSE stained cells are represent non-specific lysis (%).

### 3.10. Analysis Intracellular Cytokine by Flow Cytometry (FCM)

The mice were sacrificed on Day 14 after the second immunization, the spleens removed and splenocytes suspensions at 0.5 × 10^6^ cells/200 μL were prepared. The splenocytes were stimulated in 96-well plates with FMDV (5 μg/mL) in a humidified chamber at 37 °C and 5% CO_2_ atmosphere for 6 h. Subsequently, Monensin (2 μg/mL, Sigma Chemical Co.) was added for 4 h, and the mixture was then washed three times with PBS. Cells were blocked with 1 μL of Fcγ-Block (0.5 μg/mL) at 4 °C for 30 min, and then fixed by paraformaldehyde (4%) for 15 min at 4 °C. The cells were permeabilized using saponin (0.1%) for 10 min at 4 °C. followed by one washing with PBS. The cells were immunostained using IgG isotype controls, or double immunostained at 4 °C for 30 min using anti-IFN-γ-FITC and anti-CD4-PE, anti-IL-2-PE and anti-CD4-FITC, anti-IL-4-PE and anti-CD4-FITC, anti-IFN-γ-FITC and anti-CD8-PE. After three washes, the cytokine levels were measured using a FACS Calibur and analyzed by Cellquest Prosoftware (BD Bioscience). Cytokine positive cell at *y*-axis, and CD4^+^/CD8^+^ positive stained cells at *x*-axis. We selected the CD4/CD8 positive stained cells (*x*-axis) and two fluorescence (PE and FITC) double stained positive cells as the initial gate, The two fluorescence (PE and FITC) double stained cell represent the CD4^+^, or CD8^+^ cells that expressed cytokine, and the gate of two fluorescence (PE and FITC) positive cells was selected, and the percentage of double stained cell in total CD4^+^/CD8^+^ stained positive cells recorded as the cytokine expression level.

### 3.11. DCs Surface Co-Stimulatory Molecules Staining

Spleens were collected on Day 3 after the first immunization, and splenocyte suspensions (2 × 10^6^ cells/200 μL) were prepared and blocked with 2 μL of Fcγ mAb (0.5 μg/mL) at 4 °C for 30 min. Following two washes with PBS, the splenocytes were double stained using MHCII-PE and anti-CD11c-FITC, anti-CD80-PE and anti-CD11c-FITC, anti-CD86-PE and anti-CD11c-FITC, or anti-CD40-PE and anti-CD11c-FITC, and stained with isotype controls. The fluorescence intensities of the cells were analyzed using the FACS Calibur (BD Biosciences). FITC stained positive cells were represent DC cells, the PE stained positive cells were represented the cells which expressed surface molecular (MHC II, CD40^+^, CD80^+^, and CD86^+^). The PE and FITC double positive cells identified as those DCs that expressed surface molecular. The percentage of PE and FITC double positive cells in total cells were selected and recorded in each sample.

### 3.12. Statistical Analysis

Data analysis was performed using SPSS software (SPSS, Version 11.5, SPSS Inc., Chicago, IL, USA). Values were expressed as mean values ± standard deviation of the mean (S.D.) LSD’s multiple range test and Duncan were used to evaluate the difference among groups. *p* < 0.05 was considered to be statistically significant.

## 4. Conclusions

In the present study, pRCPS as an adjuvant for FMDV significantly enhanced the humoral and cellular immune responses via inducing DC maturation. This study indicated phosphorylated modification could enhance the immune-enhancing activity of RCPS. However, the precise mechanisms of how the structural phosphorylated modification affects RCPS biological activity should be further investigated.

## Figures and Tables

**Table 1:** Effects of the pRCPS on cytokine production in T cells by FACS (%).

Group	CD4^+^ IL-4	CD4^+^ IL-2	CD4^+^ IFN-γ	CD8^+^ IFN-γ
Naive	1.32 ± 0.21 ^d^	1.55 ± 0.16 ^d^	1.41 ± 0.18 ^d^	0.98 ± 0.07 ^d^
FMDV	3.35 ± 0.37 ^c^	2.98 ± 0.28 ^c^	2.17 ± 0.28 ^c,d^	2.13 ± 0.09 ^c^
FMDV + RCPS	6.31 ± 0.28 ^b^	4.02 ± 0.36 ^b^	7.19 ± 1.04 ^b^	4.19 ± 0.07 ^b^
FMDV + pRCPS	9.91 ± 0.56 ^a^	6.18 ± 0.23 ^a^	8.67 ± 1.31 ^a^	6.48 ± 0.19 ^a^
FMDV + alum	4.95 ± 0.38 ^c^	3.11 ± 0.14 ^c^	3.73 ± 0.48 ^c^	2.93 ± 0.07 ^c^
pRCPS	1.89 ± 0.24 ^d^	1.67 ± 0.17 ^d^	1.93 ± 0.39 ^d^	1.56 ± 0.31 ^d^

The summaries of percentage of IL-4 in total CD4^+^, IL-2 in total CD4^+^, IFN-γ in total CD4^+^, and IFN-γ in total CD8^+^ are shown in the Table 1. Data within a column without the same superscripts (a–d) differ significantly (*n* = 10, *p* < 0.05).

**Table 2:** Animal grouping.

Group	Vaccine	Adjuvant
Naive	Naive	
FMDV	200 μL FMD vaccine	
FMDV + RCPS	200 μL FMD vaccine	0.5 mg pRCPS
FMDV + pRCPS	200 μL FMD vaccine	0.5 mg pRCPS
FMDV + alum	200 μL FMD vaccine	200 μg alum
pRCPS		0.5 mg pRCPS

## Abbreviations

RCPS	*Radix Cyathulae officinalis Kuan* polysaccharides
CFSE	Carboxyfluorescein succinimidyl ester
DCs	dendritic cells
FACS	fluorescence activated cell sorting
FITC	Fluorescein isothiocyanate
PE	Phycoerythrin
PI	propidium iodide
IL-4	interleukin-4
IL-2	interleukin-2
IFN-γ	interferon-γ
LPS	lipopolysaccharide
ConA	Concanavalin A
IR	infrared
NK	natural killer cells
CTL	cytotoxic T lymphocyte
